# Treating refractory obsessive compulsive disorder with cathodal transcranial direct current stimulation over the supplementary motor area: a large multisite randomized sham-controlled double-blind study

**DOI:** 10.3389/fpsyt.2024.1338594

**Published:** 2024-05-17

**Authors:** Ghina Harika-Germaneau, Damien Heit, Dominique Drapier, Anne Sauvaget, Remy Bation, Armand Chatard, Damien Doolub, Issa Wassouf, Nicolas Langbour, Nematollah Jaafari

**Affiliations:** ^1^ Unité de Recherché Clinique Intersectorielle en Psychiatrie à vocation régionale du Centre Hospitalier Henri Laborit, Poitiers, France; ^2^ Université de Poitiers, Poitiers, France; ^3^ Centre de Recherches sur la Cognition et l’Apprentissage, Centre National de la Recherche Scientifique (CNRS 7295), Université de Poitiers, Poitiers, France; ^4^ HUGOPSY Network, Rennes, France; ^5^ Adult Psychiatry Department, Guillaume-Régnier Hospital, University of Rennes 1, Centre d’investigation Clinique (CIC) Inserm 1414, Rennes University Hospital, Rennes, France; ^6^ Nantes Université, CHU Nantes, Mouvement, Interactions, Performance, MIP, UR 4334, Nantes, France; ^7^ Université Lyon 1, Lyon University, Villeurbanne, France; ^8^ INSERM U1028, CNRS UMR5292, PSYR2 Team, Lyon Neuroscience Research Center, Lyon, France; ^9^ Psychiatric Unit, Wertheimer Neurologic Hospital, Bron, France; ^10^ Centre Hospitalier Nord Deux-Sèvres, Service de Psychiatrie Adulte, Thouars, France

**Keywords:** obsessive-compulsive disorder, supplementary motor area, treatment, tDCS, refractory

## Abstract

**Background:**

The present study evaluated the therapeutic efficacy and tolerability of 10 transcranial direct current stimulation (tDCS) sessions in treatment-resistance obsessive-compulsive disorder (OCD) patients using a multisite double-blind sham-controlled design.

**Methods:**

Eighty treatment-resistance outpatients suffering from obsessive-compulsive disorder were randomized to receive either active or sham transcranial direct current stimulation. The cathode was positioned over the supplementary motor area and the anode over the right supraorbital area. Patients were evaluated at baseline, end of treatment (day 14), one-month follow-up (day 45), and three-month follow-up (day 105) on the Yale-Brown Obsessive Compulsive Scale.

**Results:**

Although a significant interaction between time and treatment was observed, the primary endpoint—measuring the change in Yale-Brown obsessive compulsive scale scores after two weeks—was not achieved. Conversely, the secondary endpoint, which concerned the change in Yale-Brown obsessive compulsive scale scores after three months, was successfully met. It is important to note, however, that there were no significant differences in the percentage of responders and remitters at any of the post-treatment assessments. This suggests that the treatment may not have had a clinically relevant impact. Patients well received the transcranial direct current stimulation treatment, indicating its good tolerability.

**Conclusion:**

This is the largest controlled trial using transcranial direct current stimulation in treatment-resistance obsessive-compulsive disorder patients. Our results indicate the importance of studying the placebo effect in transcranial direct current stimulation and the necessity to consider a long follow-up time to best evaluate the effects of the intervention.

**Clinical trial registration:**

ClinicalTrials.gov, identifier NCT03304600.

## 1 Introduction

Despite the improvements in psychotherapy and pharmacological treatments for psychiatric diseases management over the past decade, many patients remain resistant to these treatments. Therefore, developing alternatives to classical therapies may be helpful, and neuromodulation techniques offer this promising alternative.

Non-invasive brain stimulation (NIBS) techniques are a well-tolerated, non-invasive physical therapy method that can modulate brain function. Two of the most commonly used NIBS techniques are repetitive transcranial magnetic stimulation (rTMS) and transcranial direct current stimulation (tDCS).

tDCS is becoming an increasingly important neurostimulation technique with many advantages such as ease of use, good tolerance and low cost. It involves applying a direct electric current across two flat and large electrodes placed over a targeted cortical region. This has encouraged the development of several clinical trials for its use in the management of psychiatric disorders (1) such as depression, addiction, craving, auditory verbal hallucinations in schizophrenia and more recently in obsessive compulsive disorder (OCD).

The prevailing neurobiological model of OCD implicates dysfunctional cortico-striato-thalamo-cortical (CSTC) circuits, including the medial prefrontal cortex [specifically the supplementary motor area (SMA)], anterior cingulate cortex, orbitofrontal cortex (OFC), and basal ganglia (2, 3). Among these regions, the OFC (4, 5) and the SMA (6, 7) appear to be particularly relevant based on numerous neuropsychological and neuroimaging studies.

The hypotheses of neurophysiological abnormalities proposed to explain OCD make tDCS a natural candidate for its treatment. In fact, the association of serotonin reuptake inhibitors (SRIs) and cognitive-behavioral therapy (CBT) is the usual treatment for OCD (8). Despite the improvement in pharmacological and behavioral treatments, 40 to 60% of OCD patients do not achieve satisfactory outcomes. Therefore, developing alternatives to classical therapies may be necessary (9).

The first case study using tDCS in OCD patients was published in 2013 by the team of Volpato et al. (10),. Following this pioneering publication, several other studies were conducted with a significant heterogeneity in terms of stimulation site, as well as other stimulation parameters such as electrode sizes, number of sessions and clinical characteristics of patients.

Between 2013 and 2022, 22 studies [as reviewed by (11)] have been published evaluating the therapeutic effect of tDCS on OCD. However, very few of these studies were randomized controlled trials (RCT) [For meta-analyse (12)]. Several brain regions have been targeted, including the dorsolateral prefrontal cortex, the orbitofrontal cortex (OFC), the supplementary motor area (SMA), and the right cerebellum. The choice of target was based on the dominant neurobiological model of OCD, which suggests that dysfunctional cortico–striato–thalamo–cortical (CSTC) circuits play a role in the etiology of clinical symptoms (2).

Our team recently conducted an open-label study on the use of tDCS in treating OCD (13). Twenty-one treatment refractory outpatients with OCD received 10 sessions of tDCS, with each treatment consisting of 2 mA (milliampere) of stimulation for 30 minutes. The cathode was positioned over the SMA, and the anode was placed over the right supraorbital area. Our hypothesis posits that targeting the SMA with cathodal tDCS, coupled with anodal tDCS over the right supraorbital area, may diminish obsessive and compulsive symptoms by modulating neuronal activity within the orbitofronto-striato-pallido-thalamic loop. Patients were evaluated at baseline, at the end of treatment (day 14), one month after treatment (day 45), and three month after treatment (day 105). This open label pilot study demonstrated the efficacy and safety of this tDCS protocol in treating treatment- refractory OCD patients. Despite these interesting results, two major limitations of this study could be addressed: the small sample size and the absence of a control condition. To address these limitations, we conducted a large multisite double-blind randomized clinical trial with the same tDCS parameters to confirm the efficacy and safety of this protocol in treating treatment-refractory OCD patients.

## 2 Methods

### 2.1 Overview

The trial was conducted in four specialized hospitals in France (Poitiers, Nantes, Rennes and Lyon). Ethical clearance was obtained from the Institutional Review Board of CPP Sud Est V (Approval number: 17-LABO-01), and trial registration was completed with the Clinical Trial Registry before the start of the study (ClinicalTrials.gov Identifier: NCT03304600). All patients provided written informed consent after a full description of the study and potential tDCS adverse effects.

### 2.2 Study design and randomization

We conducted a 2-weeks randomized, multisite, sham-controlled, double blind, parallel-group trial that compared the effect of tDCS on OCD symptoms. The study was conducted between November 2017 and February 2022.

Eligible patients were randomly allocated (1:1) to either active tDCS or sham tDCS stimulation using a computed based randomization. When the investigator performs randomization, after verifying the patient’s eligibility, they log in to the study’s electronic Case Report Form (eCRF). The investigator completes the “randomization” page after confirming all patient eligibility criteria. The eCRF immediately communicates the patient’s randomization number. The NeuroConn Plus stimulation system features a specific programming mode for stimulation parameters intended for clinical studies. This system is designed for double-blind studies as it provides limited user access and allows pre-programming of stimulation parameters. Selection of the stimulation type (sham/active) is done using a numerical code (5-digit), which codes for either a sham or active session; in both cases, the information displayed during the session is identical. This system ensures blinding of the experimenter. By knowing the 5-digit code, the care provider cannot determine whether the patient is receiving active or sham stimulation. To ensure that the experimenters and participants remained blinded to the tDCS condition, a commercially available sham procedure of the tDCS device was used. The care provider was required to enter a pre-programmed code that delivered either active or sham tDCS, with no knowledge of which condition the code applied to. A researcher who was not involved in tDCS delivery, data collection, or analyses established the list of codes to ensure impartiality.

To ensure the blinding of the care providers, investigators, and outcome assessors, they were all kept unaware of the treatment assignment. A blinding guess rate was not assessed in this study.

### 2.3 Participants

**Figure 1:**
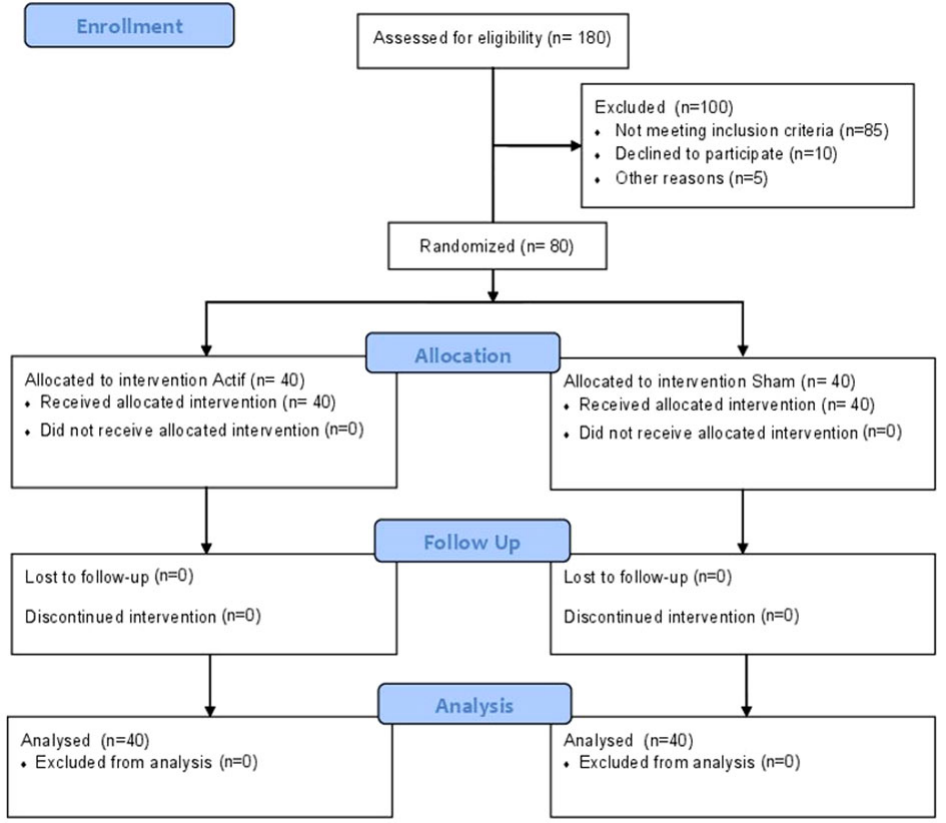
Flow diagram.

Eighty outpatients aged between 18 and 70 years with DSM-IV-TR OCD, diagnosed using the Mini-International Neuropsychiatric Interview (MINI) (14), were enrolled in the study (see **Figure 1** for the flow diagram). All patients were outpatients recruited from specialized consultations for OCD at each center.

To be eligible, patients were required to have a total score of 21 or more on the Yale Brown Obsessive Compulsive Scale (Y-BOCS), a disease duration of at least two years, and to have received at least 12-week treatments with Serotonin Reuptake Inhibitors (SRIs) and Cognitive Behavioral Therapy (CBT) without responding (treatment refractory). The current medication regimen was maintained throughout the treatment and follow-up visits. Benzodiazepines were also maintained at the same dose throughout the study.

We excluded participants who met any of the following criteria: a diagnosis of schizophrenia, current major depressive disorder, other psychotic disorders, bipolar disorder, substance and alcohol dependence within the last six months, a score of three or more on the Montgomery-Asberg Depression Rating Scale (MADRS) suicide item, moderate or severe stage in MINI, severe or unstable medical conditions, the presence of metallic implants or a history of epilepsy.

The participants did not receive any financial compensation for their participation in this study.

### 2.4 Intervention

Each patient received a total of 10 tDCS sessions, delivered once a day, 5 days a week. Stimulation sessions were delivered using a neuroConn DC stimulator (Ilmeneau, GmbH). The stimulator was connected to two rubber electrodes (7 x 5 cm, 35 cm2) placed inside a sponge, which, in turn, was soaked on each side in a saline solution (0.9% NaCl) and fixed over the sites of interest with a tubular net bandage. A typical session of tDCS consisted of delivering a direct current of 2 mA (current density of 0.57 A/m^2^) for 30 minutes. The choice of these parameters is associated with a better safety profile and is consistent with the majority of clinical studies on tDCS in OCD (11).

Electrodes were positioned on the scalp following the international 10-20-electrode placement system. The cathode was placed on the sagittal midline at 15% of the distance between inion and nasion anterior to Cz, using the international 10-20 EEG (Electroencephalogram) system to target the bilateral SMA (15). The anode was placed over the right orbitofrontal area above FP2, according to the 10-20 international system for EEG. During the tDCS session, patients were instructed to relax and stay awake with open eyes.

In the sham condition, the same stimulation parameters were displayed as in the real condition. However, after 30 seconds of real stimulation at 2 mA, brief current pulses of 110 μA (microampere) over 15 milliseconds were delivered every 550 milliseconds for the remainder of the 30-minute period. This design was intended to mimic the initial sensation of tDCS while not providing the actual stimulation.

### 2.5 Assessment

All assessments included the Y-BOCS and Y-BOCS check list, Clinical Global Impressions-Severity (CGI-S), Clinical Global Impressions-Improvement (CGI-I), Montgomery-Asberg Depression Rating Scale (MADRS), Brown Assessment of Beliefs Scale (BABS), Brief Anxiety Scale (BAS), Hospital Anxiety and Depression scale (HAD), Global assessment of Functioning (GAF) and Sheehan Disability Scale (SDS). Patients were assessed at baseline, post-tDCS treatment (14 days after baseline), after 1-month follow-up (45 days after baseline), and 3-month follow-up (105 days after baseline).

The safety of tDCS was assessed after each tDCS session using a structured interview (16).

### 2.6 Outcome measures

The primary objective of this clinical trial was to compare the change in Y-BOCS scores between the two treatment groups from baseline to day 14. The secondary outcome measures included the change in Y-BOCS scores between the two treatment groups from baseline to day 45 and day 105, respectively. We also examined responder status between the two groups from baseline to follow-up visits.

Other secondary outcomes and side effects, which are not reported in this paper but are available in supplementary online materials (SOM), include changes in various measures such as MADRS, BABS, BAS, CGI-S, CGI-I, HAD, GAF and SDS.

### 2.7 Statistics

Statistical analyses were performed using Jamovi version 2.2 (https://www.jamovi.org) and the software R, version 4.1.0 (nlme, sjplot packages; R Foundation for Statistical Computing, Vienna, Austria).

We compared the demographic and clinical characteristics at baseline between the active tDCS group and sham tDCS group using independent sample t-tests (two-tailed) and chi-square tests. Primary and secondary outcomes were analyzed using a series of linear mixed-effects models with time (baseline vs follow-up: day 14, day 45 or day 105), group (active tDCS, sham tDCS) and their cross-level interaction as independent variables. A normality test was conducted using the Kolmogorov-Smirnov test, which indicated that the data follow a normal distribution. A significance threshold of *p* < 0.05 was chosen for all tests.

## 3 Results

### 3.1 Participants

Eighty patients were randomized, with 40 patients in each group (**Figure 1**). A power analysis indicates that this sample size had adequate statistical power (1 - β = 80%) to detect significant changes in Y-BOCS scores from baseline to post-treatment assessments between the two treatment groups, assuming a medium effect size (Cohen’s d = 0.55). Both groups are comparable at baseline except for the BABS score. Insight was assessed using the BABS, which can categorizes patients into four groups based on the total score: excellent (0–3), good (4–7), fair (8–12), and poor (13–17), or a total score of 18 and a score of 0–3 on the conviction item. Many studies (17, 18) consider a score of “14 or more” as indicating poor insight. At baseline, there is a statistically significant difference between the sham and active groups. However, this difference is not clinically significant, as the score in both groups remains largely < 14.

All patients completed the 10 stimulation sessions and the evaluation visits. **Table 1** summarizes the sample characteristics of the study participants at baseline.

**Table 1:** Baseline demographic and clinical characteristics of the participants.

	Sham tDCS	Active tDCS	p-value
	**(N=40)**	**(N=40)**	
Gender (M/F)	20/20	23/17	0.501^#^
Age (years)	41.1 (11.5)	43.0 (12.7)	0.497^*^
Age at onset (years)	18.8 (9.96)	21.1 (11.2)	0.324^*^
Duration of illness (years)	14.4 (10.7)	12.8 (11.4)	0.526^*^
Y-BOCS score			
Total	27.9 (4.10)	28.7 (4.70)	0.434^*^
Compulsion	14.2 (2.38)	14.1 (2.77)	0.931^*^
Obsession	13.7 (2.42)	14.6 (2.73)	0.156^*^
CGI-S score	5.44 (0.641)	5.29 (0.802)	0.378^*^
MADRS score	11.8 (6.26)	11.8 (6.86)	0.973^*^
GAF score	48.2 (9.72)	47.1 (9.51)	0.626^*^
BAS	12.7 (6.22)	12.9 (5.09)	0.858^*^
BABS score	3.95 (3.15)	5.76 (4.25)	0.036^*^
HAD score	20.6 (7.99)	20.5 (8.44)	0.968^*^
SDS score	19.44 (6.42)	21.33 (6.33)	0.218^*^

Data are presented as mean (SD). Y-BOCS, Yale Brown Obsessive Compulsive Disorder Scale; CGI-S, Clinical Global Impressions Severity; MADRS, Montgomery-Asberg Depression Rating Scale; BAS, Brief Anxiety Scale; GAF, Global Assessment of Functioning; BABS, Brown Assessment of Beliefs Scale; HAD, Hospital Anxiety and Depression scale; SDS, Sheehan Disability Scale. *The p-value was obtained by a two sample two tailed t-test.

^#^The p-value was obtained using a Pearson c2 two-tailed test.

Patients’ treatments were maintained throughout the study and were at adequate and stable doses for at least 12 weeks before randomization. No treatment changes were reported until the end of the study (day 105).

Overall, tDCS treatment was well tolerated. There were no major clinical or cognitive side effects, all side effects were mild, short-lived, well tolerated, and spontaneously resolved.

### 3.2 Primary outcome

**Figure 2:**
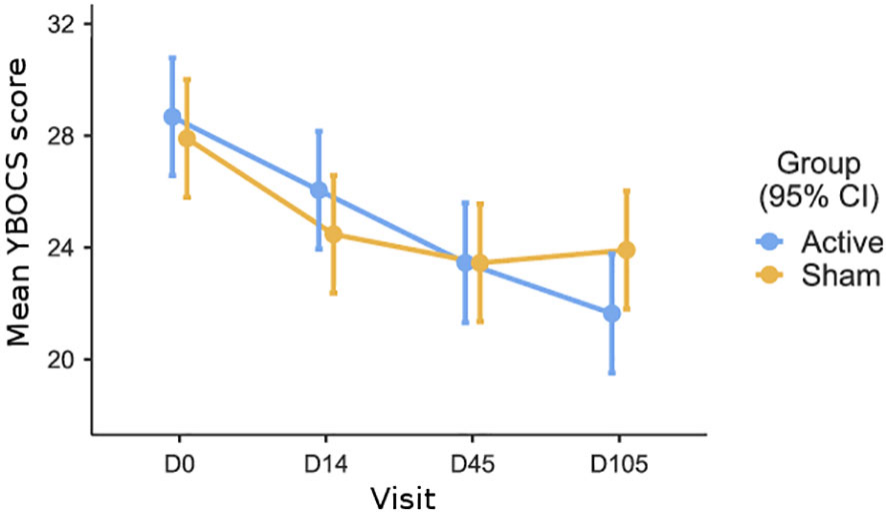
Mean Y-BOCS scores for both treatment groups (Active and Sham) at four different time points: baseline (D0), end of treatment (D14), one-month follow-up (D45), and three-month follow-up (D105).

**Figure 2** shows the mean Y-BOCS scores for both treatment groups at three different time points.

To begin with, a 2 (treatment: sham vs. tDCS) x 4 (time assessment: baseline vs. day 14 vs. day 45 vs. day 105) analysis of variance (ANOVA), with repeated measures on the last factor, was run to test the interactive effect of the treatment over time. In this analysis, there was a main effect of time (F(3,219)=30.14; p<0.001, η2 = .093), no effect of the treatment (F(1,73)=0.128; p=0.721, η2 = .001), but a significant time by treatment interaction was observed (F(13,219)=3.55; p=0.015, η2 = .011). This significant interaction indicates that the effect of the treatment varies over time.

A linear mixed model with repeated measures was used to test the primary outcome, with treatment (sham vs. tDCS) and Y-BOCS assessment (baseline vs. day 14) as factors. The results indicate a significant effect of the repeated factor (F (1,78)=30.87; p<0.001, η2 = .28), indicating that Y-BOCS scores decreased significantly over time, regardless of treatment. There was no significant overall effect of treatment (F (1,78)=1.09; p=0.29, η2 = .01), and contrary to our expectation, no significant interaction between treatment and Y-BOCS assessment (F (1,78)=0.54; p=0.46, η2 = .00). These results suggest that the change in Y-BOCS scores from baseline to the primary endpoint (day 14) did not differ significantly between the two groups.

### 3.3 Secondary outcomes

For the first secondary outcome, a linear mixed model was used with treatment (sham vs. tDCS) and Y-BOCS assessment (baseline vs. day 45 follow-up) as factors. The results indicate a significant effect of the repeated factor (F (1,75)=42.75; p<0.001, η2 = .36), showing that Y-BOCS scores decreased significantly over time, regardless of treatment. There was no significant overall effect of treatment (F (1,75)=0.56; p=0.81, η2 = .01), and, contrary to our expectation, no significant interaction between treatment and Y-BOCS assessment (F (1,75)=0.32; p=0.57, η2 = .00). In summary, Y-BOCS scores decreased significantly over time in both groups, but there was no significant difference in the reduction of Y-BOCS scores between the tDCS and sham groups at the 1-month follow-up, as indicated by the lack of significant treatment-by-assessment interaction.

For the second secondary outcome, we used a linear mixed model with repeated measures, with treatment (sham vs. tDCS) and Y-BOCS assessment (baseline vs. day 105) as factors. The results indicate a significant effect of the repeated factor (F (1,75)=59.08; p<0.001, η2 = .44), showing that Y-BOCS scores decreased significantly over time, regardless of treatment. There was no significant overall effect of treatment (F (1,75)=0.55; p=0.45, η2 = .00). However, as expected, the interaction between treatment and Y-BOCS assessment was significant (F (1,75)=4.60; p=0.035, η2 = .058), indicating that after three months, the reduction in Y-BOCS scores was significantly larger in the tDCS group than in the sham group.

Patients were classified as responders if they showed at least a decrease of 35% on the Y-BOCS and a score of 2 or less on the CGI-I (19). At day 14, 7.9% of patients were responders in active group and 15% in the sham group. At day 45, 8 patients (22.2%) were responders in active group and 5 in the sham group (12.8%). At day 105, 8 patients (22.2%) were responders in active group and 5 in the sham group (12.8%). No differences in responder status between the two groups were found at day 14 (χ^2^ = 0.964; p=0.326), at day 45 (χ^2^ = 1.15; p=0.283) and day 105 (χ^2^ = 0.929; p=0.335).

Remission is indicated as a score of ≤12 on the Y-BOCS plus CGI-S rating of 1 or 2 (19). At day 45 2 patients (both in sham group) are considered as remitted and 2 patients (one in active and one in sham group) at day 105.

## 4 Discussion

We present the largest controlled randomized trial on the use of tDCS in OCD to date. Our study employed a rigorous and well-controlled protocol with a large sample of treatment-refractory OCD patients, assessing both short and long-term effects of tDCS. This approach aligns with recommended protocols for evaluating treatment response in OCD patients (20).

tDCS is safe and well-tolerated, as represented by the low incidence of adverse events. tDCS was applied as add-on treatment to patients with a history of treatment refractory OCD, who remained on stable treatment throughout the study. The results demonstrated a significant decrease in Y-BOCS scores over time, regardless of treatment. At both the primary endpoint (day 14) and the first secondary endpoint (day 45), there was no significant difference between the sham and tDCS groups. However, at the second secondary endpoint (day 105), a significant interaction between treatment and Y-BOCS assessment emerged, indicating a more substantial reduction in Y-BOCS scores in the tDCS group compared to the sham group.

Of course, this positive finding should be considered with caution, as it might be spurious, being the sole significant effect found in favor of the treatment in this study. However, this effect, which aligns with our expectations, was clearly hypothesized in our clinical trial. If reliable, it might indicate that the placebo effect fades over time, while the effect of active stimulation persists. Notably, no differences in responder and remitter status were observed between the two groups at day 14, day 45 and day 105. It’s important to note, however, that our study was adequately powered to detect a significant effect of half a standard deviation on the Y-BOCS scale, but it was underpowered to detect larger effects that may have greater clinical relevance.

Despite the delayed effect in the reduction in Y-BOCS scores at day 105 our trial fail to demonstrate a difference between active and sham stimulation. It is noteworthy that patients in the sham group showed an improvement in their OCD symptoms over time. This is a very interesting point that is challenging to interpret, especially in the context of treatment refractory patients.

Several factors can contribute to the placebo effect. These include the patient’s belief in the treatment, their trust in the healthcare provider, the ritualistic aspects of the treatment procedure, and the overall therapeutic context, especially for patients seeking a new approach to care after several unsuccessful attempts. The patient’s expectation of improvement can trigger changes in their neurophysiology and emotional state, which may contribute to a reduction in OCD symptoms (21). Another explanation lies in the evaluation scales, according to (Mohamadi et al., 2022) (22) clinician-rated measure showed a subtantial placebo effect compared to self-reported measure. In addition, the neurobiological effect of sham tDCS remain under-addressed issue (23) with the possibility of biological effects of sham tDCS beyond the intended transient sensations.

On the other hand, the use of technologies such as non-invasive brain stimulation techniques could provide greater placebo effects compared to other treatments (24). Several randomized controlled trials (RCTs) have demonstrated a robust placebo effect of rTMS in various neuropsychiatric disorders [(25) (22, 26, 27)]. This placebo effect was also described in a recent RCT (28), where tDCS failed to demonstrate significant improvements in OCD symptoms compared to sham stimulation. Lastly, recent meta-analyses on placebo effects in OCD have indicated that compared to the intervention’s effect size, placebos are about 50% effective (22). The results of our large-scale RCT reflect these considerations and suggest that placebo effects may have been underestimated in previous open-label trials on tDCS in OCD.

While tDCS has shown promise in treating various psychiatric conditions (1), its efficacy in OCD appears to be limited. Indeed, since the first open-label studies evaluating tDCS in OCD, only a few randomized controlled trials have been published until 2023 (28–30) with mixed results. This suggests that tDCS may not be an effective standalone treatment for OCD. One potential explanation is that OCD is a complex disorder with various symptoms subtypes (31) and underlying neural mechanisms. Furthermore, treatment refractory OCD patients may exhibit distinct patterns of brain abnormalities compared with non-resistant patients (32). Also, according to the a recent meta-analysis of Pellegrini et al. (33), rTMS is effective in OCD, but primarily for those not resistant to Selective Serotonin Reuptake Inhibitor (SSRI) or failing to respond to only one SSRI trial. Overall, it’s likely that tDCS does not have a uniform effect on the brain circuits involved in OCD patients, given the variability in symptoms dimensions and treatment resistant, making it challenging to identify a universally effective tDCS protocol.

Overall, tDCS is an interesting technique in the management of OCD and its subtypes, as well as other psychiatric conditions. However, several parameters still need to be defined, including the number of sessions, the intensity of stimulation, and the placement of electrodes. Based on our results, two key considerations for future clinical trials are the importance of the placebo effect in neurostimulation studies and the importance of conducting short and long term evaluation to comprehensively document clinical outcomes.

Additionally, the long-term effects of tDCS in OCD are not well understood. Some studies have found that tDCS may produce short-term symptom improvements, but the benefits tend to fade over time. However, our results show the opposite, with a greater effect at a later stage. In pharmacological OCD treatment, patients are expected to show a measurable response only after 8-12 weeks of treatment onset (34). Consequently, similar to pharmacological and CBT treatments, neuromodulation trials in patients with OCD must consider a long follow-up time to best evaluate the effects of the intervention, rather than expecting an acute effect. In future RCT trials, it would be interesting to include an evaluation beyond 3 months to specify the long-term effect of tDCS on OCD symptoms.

It is important to note that our study has several limitations that may have affected the efficacy of the treatment. First, we conducted only 10 tDCS sessions, which may be insufficient to induce a sustained clinical effect. Kumar et al. (35) using a similar montage as ours but with 20 tDCS sessions, reported an 80% decrease in the Y-BOCS in an open-label study. Therefore, a larger number of sessions can be suggested for future studies, however, this point must be considered with caution because the Kumar study is an open-label trial. Second, our electrode montage may not be optimal, which could have contributed to the lack of efficacy in the active group. As suggested in our open-label trial (13), the clinical studies conducted to date in OCD do not provide conclusive evidence regarding the type of stimulation to be applied over the SMA. In fact, recent tDCS studies have reported improvements in obsessive-compulsive symptoms with cathodal stimulation of the SMA, while placing the anode positioned extracephalically over the lateral surface of the patient’s deltoid (36, 37). Interestingly, the clinical effects of tDCS may not rely on polarity. Gowda et al. (2019) (30) demonstrated that anodal stimulation of the SMA and cathodal stimulation over the right supraorbital area effectively treated OCD patients resistant to serotonin reuptake inhibitors (SRIs). Thus, the effects of tDCS may appear at both the anode and cathode, as well as between the electrodes, thereby influencing excitability across a broader cortical area. In our study, the anode electrode positioned over the right supraorbital area served as an “inert” reference electrode. It is plausible that the clinical effects of tDCS could also be attributed to the anodal impact on the right supraorbital area. This observation may explain intriguing clinical outcomes observed with the same electrode placement but with opposite polarity for example Mondino et al. (2015) (38), in a case report, demonstrated that cathodal stimulation over the left OFC and anodal stimulation over the contralateral occipital region alleviated symptoms in patients with treatment-resistant OCD (26% reduction in Y-BOCS). Third, in our study, patients were asked to relax and stay awake with open eyes during the tDCS session. Therefore, combining non-invasive brain stimulation with symptom provocation (39) or cognitive behavioral therapy techniques could potentially improve outcomes (40). Fourth, we did not include a blinding guess rate in order to assess the effectiveness of our blinding procedures and we use only clinician-rated measures to evaluate OCD symptoms. Finally, electrode positioning was chosen based on a standardized EEG head model, which may create a lack of specificity. These limitations should be taken into account when interpreting the results of our study and when designing future trials.

Despite the limitations, our study has several strengths. Firstly, it included the largest number of patients to date in a randomized controlled trial evaluating the use of tDCS for OCD treatment. Additionally, the multicenter design, computer-based allocation, and long follow-up period ensured a balanced distribution of factors likely to influence the response to treatment and reduced the risk of bias. Lastly, the very low attrition rate enhances the reliability of our findings.

In conclusion, the results of this clinical trial suggest that our tDCS protocol was not effective in reducing OCD symptoms. However, the low cost of tDCS and its tolerability profile point to the need for further studies evaluating its application and effectiveness in OCD patients. To date, the lack of standardized protocols makes it difficult to compare results and draw definitive conclusions. These negative results should not discourage further exploration but rather guide us toward better understanding the complexities of OCD. It is important to conduct large-scale, well-designed clinical trials to establish the true efficacy of different tDCS protocols in OCD. By addressing the limitations and building upon the existing knowledge, we can gain a clearer understanding of how to maximize the potential of tDCS in the treatment of OCD.

Following the results of our study, several elements can be considered for future clinical trials. Among these points, we can mention the number of tDCS sessions; the necessity of maintaining long-term evaluations, the association of stimulations with a symptom personalized symptom provocation, as well as the choice of stimulation areas. Future trials could benefit from incorporating neuroimaging and/or neurophysiological data, as well as numerical simulation techniques, in addition to clinical assessments to better understand the neurobiological mechanisms underlying tDCS. Such studies could shed light on optimal electrode placement and stimulation parameters, as well as the ideal number of treatment sessions required to produce a sustained clinical effect.

## Data availability statement

The original contributions presented in the study are included in the article/**Supplementary Material**. Further inquiries can be directed to the corresponding author.

## Ethics statement

The studies involving humans were approved by Institutional Review Board of CPP Sud Est V (Approval number: 17-LABO-01). The studies were conducted in accordance with the local legislation and institutional requirements. The participants provided their written informed consent to participate in this study.

## Author contributions

GH-G: Conceptualization, Data curation, Formal analysis, Investigation, Methodology, Project administration, Supervision, Validation, Writing – original draft, Writing – review & editing. DH: Conceptualization, Data curation, Funding acquisition, Resources, Writing – original draft. DoD: Investigation, Visualization, Writing – original draft. AS: Data curation, Investigation, Visualization, Writing – review & editing. RB: Investigation, Supervision, Visualization, Writing – review & editing. AC: Methodology, Supervision, Writing – review & editing. DaD: Investigation, Writing – review & editing. IW: Investigation, Visualization, Writing – review & editing. NL: Conceptualization, Data curation, Formal analysis, Funding acquisition, Methodology, Visualization, Writing – original draft. NJ: Funding acquisition, Investigation, Methodology, Project administration, Resources, Supervision, Validation, Visualization, Writing – review & editing.
