# Supplementary material for: Treating refractory obsessive compulsive disorder with cathodal transcranial direct current stimulation over the supplementary motor area: a large multisite randomized sham-controlled double-blind study
Source: Front Psychiatry. 2024 May 17;15:1338594. doi: 10.3389/fpsyt.2024.1338594 (PMC11140596; doi:10.3389/fpsyt.2024.1338594)
Supplement: Supplementary file 1 [file Table_1.docx]

Supplementary material

## Treating Refractory Obsessive-Compulsive Disorder with cathodal Transcranial Direct Current Stimulation over the SMA: a large multi randomized sham controlled trial

Summary

Table S1: Common side effect between in Active tDCS group and Sham tDCS group

Table S2: Primary and secondary outcomes between Active tDCS group and Sham tDCS group

Table S3: Summary of linear mixed-effects models for CGI-S, MADRS and HAD scales

Table S4: Summary of linear mixed-effects models for BAS and BABS scales

Table S5: Summary of linear mixed-effects models for SDS

Table S6: Summary of follow-ups repeated measures

Table S1: Side effect between in Active tDCS group and Sham tDCS group

|  | Active (%) | Sham (%) |
| --- | --- | --- |
| Mild tingling sensation | 56.3 % | 49.8% |
| Skin redness | 36.3% | 19.8 % |
| Burning sensation | 20.5% | 8.3% |
| Sleepiness | 16.3 % | 25.8% |
| Itching | 25.3 % | 6.8 % |
| Headache | 9 % | 11.3% |
| Scalp pain | 2.5 % | 1% |
| Trouble concentrating | 2.3% | 2.3% |
| Neck pain | 1.3 % | 1.8% |
| Acute mood change | 0 % | 2.5% |

Table S2: Primary and secondary outcomes between Active tDCS group and Sham tDCS group

|  |  | Active tDCS | | | |  | Sham tDCS | | | |
| --- | --- | --- | --- | --- | --- | --- | --- | --- | --- | --- |
|  |  | D0 | D14 | D45 | D105 |  | D0 | D14 | D45 | D105 |
| Scales |  | (N=40) | (N=40) | (N=40) | (N=40) |  | (N=40) | (N=40) | (N=40) | (N=40) |
| Y-BOCS |  | 28.7 (4.70) | 26.1 (6.16) | 23.3 (8.49) | 21.6 (8.05) |  | 27.9 (4.10) | 24.5 (6.92) | 23.5 (7.50) | 24.1 (6.80) |
| *Missing* |  | *0 (0%)* | *0 (0%)* | *3 (7.5%)* | *2 (5.0%)* |  | *0 (0%)* | *0 (0%)* | *0 (0%)* | *1 (2.5%)* |
|  |  |  |  |  |  |  |  |  |  |  |
| CGI-S |  | 5.29 (0.802) | 5.00 (0.838) | 4.80 (0.994) | 4.66 (1.17) |  | 5.44 (0.641) | 5.05 (0.959) | 4.82 (1.45) | 5.08 (1.06) |
| *Missing* |  | *2 (5.0%)* | *2 (5.0%)* | *5 (12.5%)* | *2 (5.0%)* |  | *1 (2.5%)* | *0 (0%)* | *1 (2.5%)* | *1 (2.5%)* |
|  |  |  |  |  |  |  |  |  |  |  |
| MADRS |  | 11.8 (6.86) | 10.3 (9.56) | 11.4 (9.80) | 12.0 (8.05) |  | 11.8 (6.26) | 11.4 (8.37) | 12.1 (8.62) | 11.7 (7.01) |
| *Missing* |  | *1 (2.5%)* | *0 (0%)* | *3 (7.5%)* | *2 (5.0%)* |  | *1 (2.5%)* | *0 (0%)* | *2 (5.0%)* | *1 (2.5%)* |
|  |  |  |  |  |  |  |  |  |  |  |
| BAS |  | 12.9 (5.09) | 10.2 (6.24) | 10.6 (7.87) | 11.3 (7.97) |  | 12.7 (6.22) | 10.7 (7.65) | 10.5 (7.43) | 11.2 (5.99) |
| *Missing* |  | *1 (2.5%)* | *0 (0%)* | *3 (7.5%)* | *2 (5.0%)* |  | *1 (2.5%)* | *0 (0%)* | *2 (5.0%)* | *1 (2.5%)* |
|  |  |  |  |  |  |  |  |  |  |  |
| BABS |  | 5.76 (4.25) | 4.79 (3.60) | 3.84 (3.39) | 3.89 (3.59) |  | 3.95 (3.15) | 3.90 (4.15) | 3.16 (2.93) | 3.18 (2.52) |
| *Missing* |  | *2 (5.0%)* | *1 (2.5%)* | *3 (7.5%)* | *2 (5.0%)* |  | *1 (2.5%)* | *0 (0%)* | *2 (5.0%)* | *2 (5.0%)* |
|  |  |  |  |  |  |  |  |  |  |  |
| HAD |  | 20.5 (8.44) | 19.7 (8.31) | 18.2 (9.32) | 18.6 (8.66) |  | 20.6 (7.99) | 17.2 (8.19) | 17.8 (8.09) | 19.5 (9.31) |
| *Missing* |  | *0 (0%)* | *0 (0%)* | *3 (7.5%)* | *2 (5.0%)* |  | *0 (0%)* | *0 (0%)* | *1 (2.5%)* | *2 (5.0%)* |
|  |  |  |  |  |  |  |  |  |  |  |
| SDS |  | 21.2 (6.33) | *19.8 (6.75)* | *19.2 (7.49)* | *18.8 (7.92)* |  | *19.4 (4.62)* | *18.6 (7.24)* | *18.4 (7.79)* | *18.1 (8.28)* |
| *Missing* |  | *1 (2.5%)* | *0 (0%)* | *3 (7.5%)* | *2 (5.0%)* |  | *1 (2.5%)* | *1 (2.5%)* | *1 (2.5%)* | *4 (10.0%)* |

Data are presented as mean (SD). YBOCS: Yale Brown Obsessive Compulsive Disorder Scale; CGI: Clinical Global Impressions; MADRS: Montgomery-Asberg depression rating scale; BAS: Brief Anxiety Scale; BABS: Brown Assessment of Beliefs Scale; HAD: Hospital Anxiety and Depression scale; SDS : Sheehan Disability Scale

Table S3: Summary of linear mixed-effects models for CGI-S, MADRS and HAD scales

|  | CGI-S score | | | MADRS score | | | HAD score | | |
| --- | --- | --- | --- | --- | --- | --- | --- | --- | --- |
| *Fixed effect Omnibus test* | *F* | *(Num df-Den df)* | *p* | *F* | *(Num df-Den df)* | *p* | *F* | *(Num df-Den df)* | *p* |
| **Baseline-D14** |  |  |  |  |  |  |  |  |  |
| Group | *0.314* | *(1–78.7)* | *0.557* | *0.0990* | *(1-78.1)* | *0.754* | *0.530* | *(1-78.0)* | *0.469* |
| Visit | *22.063* | *(1-75.3)* | ***<.001*** | *1.1651* | *(1-76.9)* | *0.284* | *10.223* | *(1-78.0)* | ***0.002*** |
| Group*Visit | *0.195* | *(1-75.3)* | *0.660* | *0.4954* | *(1-76.9)* | *0.484* | *3.975* | *(1-78.0)* | *0.050* |
|  |  |  |  |  |  |  |  |  |  |
| **Baseline-D45** |  |  |  |  |  |  |  |  |  |
| Group | *0.259* | *(1-80.0)* | *0.612* | *0.0157* | *(1-78.4)* | *0.901* | *0.0076* | *(1-78.1)* | *0.931* |
| Visit | *16.866* | *(1-77.1)* | ***<.001*** | *0.0022* | *(1-73.5)* | *0.963* | *13.6497* | *(1-75.0)* | ***<.001*** |
| Group*Visit | *0.122* | *(1-77.1)* | *0.728* | *0.0867* | *(1-73.5)* | *0.769* | *0.1138* | *(1-75.0)* | *0.337* |
|  |  |  |  |  |  |  |  |  |  |
| **Baseline-D105** |  |  |  |  |  |  |  |  |  |
| Group | *2.42* | *(1-78.9)* | *0.124* | *0.0361* | *(1-78.4)* | *0.850* | *0.132* | *(1-78.2)* | *0.718* |
| Visit | *22.42* | *(1-75.9)* | ***<.001*** | *0.0074* | *(1-75.4)* | *0.932* | *4.260* | *(1-75.2)* | ***0.042*** |
| Group*Visit | *1.90* | *(1-75.9)* | *0.172* | *0.0096* | *(1-75.4)* | *0.922* | *0.638* | *(1-75.2)* | *0.427* |
|  |  |  |  |  |  |  |  |  |  |

Table S4: Summary of linear mixed-effects models for BAS and BABS scales

|  | BAS score | | | BABS score | | |
| --- | --- | --- | --- | --- | --- | --- |
| *Fixed effect Omnibus test* | *F* | *(Num df-Den df)* | *p* | *F* | *(Num df-Den df)* | *p* |
| **Baseline-D14** |  |  |  |  |  |  |
| Group | *0.0132* | *(1-78.1)* | *0.909* | *3.28* | *(1-78.6)* | *0.074* |
| Visit | *10.2111* | *(1-77.1)* | ***0.002*** | *1.19* | *(1-76.5)* | *0.280* |
| Group*Visit | *0.3651* | *(1-77.1)* | *0.547* | *1.20* | *(1-76.5)* | *0.276* |
|  |  |  |  |  |  |  |
| **Baseline-D45** |  |  |  |  |  |  |
| Group | *0.0214* | *(1-79.1)* | *0.885* | *3.40* | *(1-75.9)* | *0.069* |
| Visit | *8.1434* | *(1-75.8)* | ***0.006*** | *10.99* | *(1-71.0)* | ***0.001*** |
| Group*Visit | *0.0011* | *(1-75.8)* | *0.973* | *2.06* | *(1-71.0)* | *0.156* |
|  |  |  |  |  |  |  |
| **Baseline-D105** |  |  |  |  |  |  |
| Group | *0.0194* | *(1-78.4)* | *0.890* | *3.92* | *(1-76.1)* | *0.051* |
| Visit | *4.2266* | *(1-76.0)* | ***0.043*** | *14.88* | *(1-71.8)* | ***<.001*** |
| Group*Visit | *0.0670* | *(1-76.0)* | *0.796* | *1.95* | *(1-71.8)* | *0.167* |
|  |  |  |  |  |  |  |

Table S5: Summary of linear mixed-effects models for SDS

|  | SDS Score | | |
| --- | --- | --- | --- |
| *Fixed effect Omnibus test* | *F* | *(Num df-Den df)* | *p* |
| **Baseline-D14** |  |  |  |
| Group | *1.138* | *(1-77.1)* | *0.289* |
| Visit | *5.100* | *(1-76.3)* | ***0.027*** |
| Group*Visit | *0.495* | *(1-76.3)* | *0.484* |
|  |  |  |  |
| **Baseline-D45** |  |  |  |
| Group | *0.681* | *(1-78.7)* | *0.412* |
| Visit | *6.623* | *(1-74.6)* | ***0.012*** |
| Group*Visit | *0.533* | *(1-74.6)* | *0.468* |
|  |  |  |  |
| **Baseline-D105** |  |  |  |
| Group | *0.423* | *(1-78.3)* | *0.517* |
| Visit | *6.913* | *(1-73.0)* | ***0.010*** |
| Group*Visit | *1.151* | *(1-73.0)* | *0.287* |
|  |  |  |  |
|  |  |  |  |

| Table S6: Summary of follow-ups repeated measures  *Within Subjects Effects* | | | | | | |
| --- | --- | --- | --- | --- | --- | --- |
|  |  |  |  |  |  |  |
|  | *Sum of Squares* | *df* | *Mean Square* | *F* | *p* | *η²* |
| *Visit* | *1376.5* | *3* | *458.84* | *30.303* | *< .001* | *0.057* |
| *Visit* **Group* | *167.8* | *3* | *55.94* | *3.694* | *0.013* | *0.007* |
| *Visit* * *SEX* | *30.0* | *3* | *9.99* | *0.660* | *0.578* | *0.001* |
| *Residual* | *3270.6* | *216* | *15.14* |  |  |  |
| *Note. Type 3 Sums of Squares* | | | | | | |

| *Between Subjects Effects* | | | | | | |
| --- | --- | --- | --- | --- | --- | --- |
|  |  |  |  |  |  |  |
|  | *Sum of Squares* | *df* | *Mean Square* | *F* | *p* | *η²* |
| *Group* | *20.7* | *1* | *20.7* | *0.153* | *0.697* | *0.001* |
| *SEX* | *36.7* | *1* | *36.7* | *0.272* | *0.604* | *0.002* |
| *Residual* | *9723.8* | *72* | *135.1* |  |  |  |
| *Note. Type 3 Sums of Squares* | | | | | | |

| *Post Hoc Comparisons - Visit* * *Group* | | | | | | | | | |  |
| --- | --- | --- | --- | --- | --- | --- | --- | --- | --- | --- |
| *Comparison* | | | | |  | | | | | |
| *Visit* | *Group* |  | *Visit* | *Group* | *Mean Difference* | *SE* | *df* | *t* | *p* | |
| *Baseline* | *Active* | *-* | *Baseline* | *Sham* | *0.401* | *0.986* | *72.0* | *0.407* | *0.685* | |
|  |  | *-* | *Day 14* | *Active* | *2.842* | *0.841* | *72.0* | *3.381* | *0.001* | |
|  |  | *-* | *Day 45* | *Active* | *5.305* | *1.104* | *72.0* | *4.806* | *< .001* | |
|  |  | *-* | *Day 105* | *Active* | *7.265* | *1.056* | *72.0* | *6.881* | *< .001* | |
|  | *Sham* | *-* | *Day 14* | *Sham* | *3.479* | *0.805* | *72.0* | *4.319* | *< .001* | |
|  |  | *-* | *Day 45* | *Sham* | *4.269* | *1.057* | *72.0* | *4.038* | *< .001* | |
|  |  | *-* | *Day 105* | *Sham* | *3.952* | *1.012* | *72.0* | *3.907* | *< .001* | |
| *Day 14* | *Active* | *-* | *Day 14* | *Sham* | *1.038* | *1.516* | *72.0* | *0.684* | *0.496* | |
| *Day 45* | *Active* | *-* | *Day 45* | *Sham* | *-0.634* | *1.852* | *72.0* | *-0.342* | *0.733* | |
| *Day 105* | *Active* | *-* | *Day 105* | *Sham* | *-2.911* | *1.727* | *72.0* | *-1.686* | *0.096* | |
